# The impact of the use of different types of gloves and bare hands for preparation of clean surgical instruments^1^

**DOI:** 10.1590/1518-8345.1127.2805

**Published:** 2016-10-10

**Authors:** Camila Quartim de Moraes Bruna, Rafael Queiroz de Souza, Irineu Francisco Silva Massaia, Áurea Silveira Cruz, Kazuko Uchikawa Graziano

**Affiliations:** 2 PhD, Visiting Professor, Faculdade de Ciências Médicas, Santa Casa de São Paulo, São Paulo, SP, Brazil.; 3 PhD, Researcher, Escola de Enfermagem, Universidade de São Paulo, São Paulo, SP, Brazil.; 4 PhD, Assistant Professor, Faculdade de Ciências Médicas, Santa Casa de São Paulo, São Paulo, SP, Brazil.; 5 PhD, Researcher, Centro de Cultura de Células, Instituto Adolfo Lutz, São Paulo, SP, Brazil.; 6 PhD, Retired Full Professor, Escola de Enfermagem, Universidade de São Paulo, São Paulo, SP, Brazil

**Keywords:** Organic Wastes, Surgical Instruments, Hand, Gloves, Protective, Toxicity, Nursing

## Abstract

**Objectives:**

to determine if there are differences on the safety of the preparation of clean surgical instruments using different types of gloves and bare hands and evaluate the microbiological load of these preparations without gloves.

**Method:**

laboratory procedure with a pragmatic approach, in which the samples were handled with different types of gloves and bare hands. In addition, cytotoxicity assays were carried out by means of the agar diffusion method. Further samples were subjected to microbiological analysis after being handled without gloves.

**Results:**

none of the samples showed cytotoxic effect. All microbiological cultures showed growth of microorganisms, but no microorganism has been recovered after autoclaving.

**Conclusion:**

there were no differences in the cytotoxic responses regarding the use of different types of gloves and bare hands in the handling of clean surgical instruments, which could entail iatrogenic risk. It is noteworthy that the use of gloves involves increase in the costs of process and waste generation, and the potential allergenic risk to latex.

## Introduction

The preparation ensures that surgical instruments are made available in the sterile field after being inspected in regard the proper cleaning and in good operating condition. It is also at this time that the instruments are separated and distributed according to the arrangement in which they will be presented in the operative field and then packaged to be subsequently routed to sterilization. 

The preparation step is performed by means of ostensible handling of surgical instruments and should consist of a thorough inspection, in order to identify residual dirt and possible mechanical failures, including all indentations and racks^1^ ^-^ ^2^. It is also recommended that the staff involved in the preparation of clean instruments wear private clothes, cap^3^, gloves and masks^4^. 

Although there is recommendation for the use of gloves in the handling performed during preparation, it is based on the theoretical deduction since no study was found in the literature to support such recommendation. In order to investigate if there are risks in the preparation of surgical instruments, this research aimed to determine if there are differences regarding safety using different types of gloves and bare hands during the inspection and arrangement of surgical instruments after cleaning, and to identify and quantify the microbial load after handling of these instruments without gloves. 

## Method

Experimental laboratory-based research with a pragmatic approach, approved by the Ethics in Research Committee of the University Hospital of the University of São Paulo -HU-USP, under the protocol CEP-HU/USP:1.264/13. The research was divided into two stages, one for cytotoxicity analysis of the samples handled using gloves and bare hands, and another for microbiological analysis of the samples handled with bare hands. 

Ophthalmic hydrodissection aluminum cannulas with about 4.0 cm long, 0.6 mm in diameter and 0.2 mm in the distal portion (Steel Inox^(r)^, Brazil) were used as samples in the cytotoxic analysis stage. For the microbiological analysis stage, brand new stainless steel surgical instruments (Anatomical non-toothed tweezers - *Erwin Guth(tm)*, Brazil) of 14 cm were used. The choice of these instruments was based on the possibility of inoculating the sample (hydrodissection cannulas) directly onto the agar layer, without causing damage to the cellular monolayer due to the weight of the sample, and inoculation of the sample (anatomical tweezers) directly in the tube containing culture medium. 

In the cytotoxic analysis stage, samples (hydrodissection cannulas) were subjected to manual cleaning with potable water, enzymatic detergent (Multi-enzymatic detergent, 3M^(r)^, Brazil) and soft bristle brush. Subsequently, the researcher prepared the samples through handling according to the group they belonged, as follows: five samples using powdered latex gloves (C1 Group), five samples using non-powdered latex gloves (C2 Group), five samples using vinyl gloves (C3 Group) and five samples using nitrile gloves (C4 Group). The Material and Sterilization Centre (CME) staff handled five samples without the use of gloves (C5 Group). The handling was characterized by the touch of hands along the entire length of the samples for 30 seconds. This time represented an average time calculated by the researcher, based on practical observation, for the inspection of complex and simple tweezers. Samples were packaged using surgical grade paper/film and sterilized in an autoclave under saturated steam pressure at 135°C for 5 minutes. Samples were then analyzed with respect to cellular toxicity by means of the agar diffusion method^5^. Based on the *United States Pharmacopeia -* USP-34^6^, the cell line used was the NCTC Clone 929 (L cell, derivative of Strain L - mouse connective tissue), cataloged in the Cell Culture Core collection of the IAL (Adolfo Lutz Institute) under the protocol number CCIAL020, derived from the *American Type Culture Collection* (ATCC^(r)^ CCL-1(tm)). *In vitro* cytotoxicity assays can be used as a first step in the assessment of biological materials. These methods are developed to determine the biological response of a cell culture when exposed to a material or extracts of it. Cytotoxicity is determined qualitatively or quantitatively by measuring a number of parameters on a scale ranging from zero to four, with grade zero representing absence of cell damage and grade four representing an expressive cell death^5^. The assays were performed within a biological safety cabinet, in a clean room with absolute filter system and positive pressure. The cell line NCTC clone 929 was grown in Eagle's Minimal Essential Medium, supplemented with 0.1 mM non-essential amino acids, 1.0 mM sodium pyruvate and 10% fetal bovine serum without antibiotics (MEM with 10% FBS). The suspended cells were plated in 5 ml into Petri plates (3.0X10^5^ cells/mL). Cells were incubated for 48 hours at 37ºC in a humidified atmosphere containing 5% CO_2_. The culture medium was discarded after formation of the cell monolayer and 5 ml of *overlay* medium were added into the Petri dishes. The *overlay* medium is composed of twice-concentrated MEM with 1.8% agar, containing 0.01% vital dye neutral red. The agar was melted and mixed with the MEM at 44?1ºC. The Petri plates were incubated once again in an incubator, in an atmosphere of 5% CO_2_ at 37ºC for 24 hours. The cannulas were deposited on the agar cell cultures in Petri dishes in order to evaluate a possible toxicity, always using sterile materials and aseptic techniques. Fragments of 0.5 cm of each type of glove (powdered latex, non-powdered latex, vinyl and nitrile) were also placed on the monolayers as control of the toxicity of the glove solely. Fragments of 0.5 cm in diameter of rubber latex were used as positive controls and fragments of 0.5 cm in diameter of nontoxic filter paper as negative controls. A negative control, in which samples were handled with tweezers, (without the touch of hands) was carried out to evaluate the toxicity. Cell toxicity was observed macroscopically through the formation of colorless halo in or around the toxic material, measured with a calibrated caliper, and microscopically by observing the changes in the morphology and cell death around the samples. All assays were performed in triplicate. The interpretation of these results was based on the biological reactivity grades of the agar diffusion method, described in ISO 10.993-5:2009 standard^5^. 

On the microbiological analysis phase, samples (anatomical non-toothed tweezers) were subjected to manual cleaning using soft bristle brush, clean water and enzymatic detergent (HS Zyme - H Strattner(tm), Brazil). After cleaning, 20 samples were handled by the CME staff, who used mask, cap and private clothes. The handling followed the same pattern established for the cytotoxic analysis step. After handling, the samples were divided into two groups. Ten samples were placed by the staff in sterile plastic bags containing 200 ml of 0.9% Saline Solution (SS - Baxter(tm), USA), which were subsequently sealed to form the M1 Group. Ten other samples were also inoculated by the staff directly into glass tubes with screw caps containing 100 ml (enough to completely submerge the tweezers) of *Tryptic Soy Broth* (TSB - BD Difco(tm), USA), forming the M2 Group. Ten other samples handled by the staff were directly placed on surgical grade paper and autoclaved at 134°C for 5 minutes, forming the sterilization Control Group. Samples were then routed for incubation and analysis. After inoculation in TSB medium, samples of the M2 Group and sterilization Control Group were incubated in an incubator (FANEM^(r)^, Brazil) at 37°C ? 2ºC for 14 days, with daily measurements to identify possible turbidity. The identification of microorganisms was carried out for the samples of the M2 Group that showed turbidity. 

The samples of the M1 Group and half of the samples of the sterilization Control Group, placed in plastic bags containing 0.9% SS, were sonicated three times in an ultrasonic washer for 5 seconds (Model USC-2800, Enge Solutions(tm), USA). Subsequently, they were shaken in an orbital shaker for 10 minutes (Model 255 - B, Fanem^(r)^, Brazil) at 160 revolutions per minute (rpm), in order to detach and eluate the microorganisms present in the samples. In a biological safety cabinet (Model VLFS 12 - VECO^(r)^, Brazil), the seals of the bags were removed with a sterilized scissor and their contents were poured into the sterilized filtration system (Sterifil^(r)^ Milipore(tm), USA), coupled to a Kitasato flask connected to a vacuum pump, using a 0.20 micron membrane (Merck Milipore(tm), USA), configuring the membrane filtration method^6^. The contents of the bags were divided in two. Therefore, each membrane filtered 100 mL of the washed microbial load resulting from the extraction of each sample, and a membrane was placed in a Petri dish containing blood agar (Probac^(r)^, Brazil), with the objective of promoting non-selective growth of aerobic microorganisms, and the other membrane was placed on agar Anaerinsol (Probac^(r)^, Brazil) to promote the growth of anaerobic microorganisms. The plates were sealed and incubated in an incubator (FANEM^(r)^, Brazil) at 37ºC ? 2ºC for 14 days, with daily measurement to ascertain microbial growth. The agar Anaerinsol plates were previously placed in anaerobic jars (Probac^(r)^, Brazil). The identification of microorganisms was carried out in the plates that showed microbial growth, by means of their morphotinctorial properties, catalase test, coagulase test, hemolysis test on blood agar and bile esculin test^7^. 

To set the size of the sample, all types of gloves available in the market for immediate use (powdered latex, non-powdered latex, nitrile and vinyl) were used in the experiment. Initially, the sample size was settled in quintuplicate for each type of glove, selecting them randomly from each box. Based on the results of this sample, it was proposed to expand the size of the sample supported by the differences that would be found. Given the absence of differences in outcomes between the two groups, it was possible to complete the survey with five samples for each group. 

## Results

None of the samples showed toxicity (Grade 2) in the cytotoxic analysis stage, regardless of the type of glove used in the handling thereof. All samples of gloves exhibited toxicity (Grade 3) when placed directly onto the cell layer. The positive controls of the cytotoxicity tests showed cell death halos (cytotoxic effect) and the negative controls did not show toxicity. The negative controls (without handling) did not show toxicity in any of the samples. 

The results of the tests of the microbiological analysis stage showed a microbial growth for all samples in the quantitative stages of M1 and in the qualitative stage of M2. In the quantitative analysis stage of the aerobic and anaerobic microorganisms, microbial growth was observed in all five samples and the microorganisms were isolated, as well as their respective loads, as described in Table 1. 

**Table 1:**
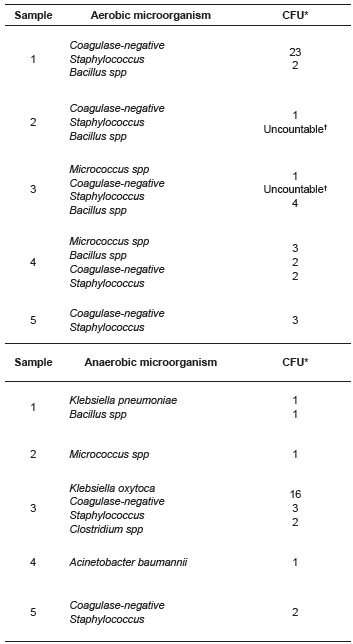
Distribution of aerobic and anaerobic microorganisms of the quantitative stage isolated from samples handled without gloves. São Paulo, SP, Brazil, 2014

*Colony forming units

†Over 300 CFU

**Figure 1:**
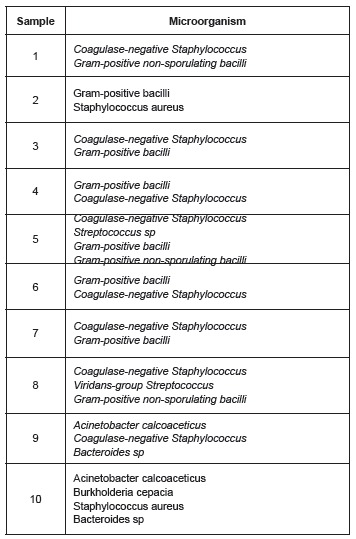
Distribution of microorganisms of the qualitative phase isolated from the samples handled without gloves. São Paulo, SP, Brazil, 2014

The microorganisms identified in the qualitative phase of the microbiological stage are described in Figure 1.

The sterilization control group did not show growth of microorganisms in any of the samples.

## Discussion

The results showed the toxicity (Grade 3) of the gloves when they were placed directly on the agar layer. Since the isolated samples showed no cytotoxicity, it was inferred that the toxicity found in the results of the samples was transferred through the handling. However, this toxicity was graded as moderate (Grade 2) and did not represent a toxicological risk to the cells. According to ISO 10.993-5:2009^5^, health products are released for use when their toxicity degree are up to Grade 2. 

Initially it was thought that there would be differences with regard to toxicity for each type of glove since it is known that latex is aggressive to the cells and is used even as a positive control in cytotoxicity assays. Nitrile gloves, which until then were considered less toxic, showed similar toxicity as the other gloves.

The handling of samples without the use of gloves showed the same degree of toxicity than the use of gloves. It was not possible to determine which element or residue provided the toxicity of the hands to the samples, representing a limitation of the study. Possibly, the endotoxins emerged, as a milder form, after sterilization of instruments. 

Endotoxins are toxins derived from cell lysis of Gram-negative bacteria and are heat stable, therefore, they are not degraded after autoclaving, which can lead to serious immune and inflammatory responses^8^. They are able to destroy the cell layer in a cytotoxicity assay. 

The microorganisms isolated from samples in the microbiological analysis stage have the human microbiota and the environment as their habitat, but some of them, such as *Burkholderia cepacia, Acinetobacter baumannii, Klebsiella pneumoniae* and *Acinetobacter calcoaceticus,* for example, can play an important role in nosocomial infections^9^.

In this study, the bacteria found were mostly similar to the bacteria isolated from the hands of health professionals as observed in other studies, as well as the number of species isolated^10^ ^-^ ^11^. Although there has not been conducted a search of anaerobic microorganisms during the qualitative phase, *Bacteroides sp* and strict anaerobic microorganisms that are part of the human microbiota were isolated^12^. This fact was a finding probably due to the anaerobic condition formed in the threaded pipes and to the large volume of medium. 

 The results showed microbial growth of about one to four colony forming units (CFU) per sample and two samples showed countless growth, which was characterized by ≥10^2^ log. Studies have shown that the microbial load of the hands of professionals involved in health care varies from 3.9 x 10^4^ to 4.6 x 10^6^ CFU/cm^2(^ ^13^. 

 Although all samples handled without gloves have been satisfactorily sterilized (which does not exclude the presence of endotoxins), these results must be considered because they indicate a possible failure in the hand hygiene of the staff involved in the preparation of instruments. 

 Concerns about issues related to quality of hand hygiene of CME staff are relevant. Adherence to this procedure ranged from 30% to 48% for health care workers involved in direct patient care^14^ ^-^ ^15^. It is assumed that this adherence is even lower in the clean area of the CME, due to the lack of direct contact with the patient and contaminated material. 

Organizations that guide the routine at CME^1^ ^,^ ^3^ ^,^ ^16^ recommend that employees who inspect the material always wash their hands when handling clean material, before and after using the toilet, eating and performing tasks other than handling clean instrumental. 

Even with the lack of growth of microorganisms in the samples after the sterilization method used, the preparation should not increase the microbial load of instruments. To establish the use of gloves due to failures in the control of hand hygiene of staff involved in the preparation is not the ideal solution, because it does not solve the problem of low adherence to hand hygiene. 

Similarly, the use of gloves with the aim to protect the employees from possible residues not eliminated by the cleaning of instruments makes it hard to solve another problem: the poor quality of the cleaning process, because regardless of how the instruments are washed, mechanical or manually, they should be left clean in the preparation area. After all, cleaning has proven successful in reducing the organic and inorganic load of instruments^17^ ^-^ ^20^, ensuring the safety in the handling of these materials when they are cleaned, without the need of the use of Personal Protective Equipment, such as gloves. 

Washing machines that complete their cycles and still leave the instruments with a smell of blood or grease or with apparent dirt, require maintenance, followed by validation and certification. Similarly, employees who perform manual cleaning of instruments and leave them dirty also need training. Therefore, the fact that the instrumental comes dirty to the preparation area would not be *a priori*, the decisive aspect for the use of gloves. 

When not recommended, the use of gloves is a waste of resources and does not contribute to the reduction of cross-transmission of microorganisms, which can also reduce the opportunities for hand hygiene. 

In Brazil, although there is evidence that latex allergy increases with occupational exposure and occupational asthma is caused exclusively by the continued use of latex gloves, their use is still significant^21^. Some authors believe that the use of latex gloves should be restricted and discouraged, particularly powdered gloves, in cases where there is no risk of exposure to contaminants^21^. 

The issue of costs must be considered, both regarding the purchase of the glove as its disposal. The data indicate that the implementation of universal precautions in a teaching hospital may represent an increase of 92% in the overall costs of the institution and the expenses with the purchasing of gloves represent two thirds of this amount^22^. No data on the cost of the use of gloves in Brazil were found. 

Another point of concern is the disposal of these gloves. It is believed that annually, 100 billion gloves are discarded in the world^23^. In Brazil, the law considers disposed gloves as A4 waste type, and therefore, not suitable for recycling^24^. 

The different types of handling of surgical instruments performed in this study were equivalent, as they have similar degrees of cytotoxicity. Therefore, from the perspective of the generation of waste resulting from the use of gloves, it is clear the recommendation of the preparation without the use of gloves, contrary to the recommendation in effect today in Brazil^4^. Therefore, increased attention must be paid to the hand hygiene during this stage of reprocessing. 

## Conclusion

In Brazil, currently, there is a recommendation for the use of gloves in the preparation of surgical instruments, however, this study demonstrated that there is no difference in the preparation regarding toxicity in the preparation with or without the use of different types of gloves. In addition, there are the disadvantages presented here with the use of gloves, such as the risk of latex sensitization of the health care professionals due to the use of the glove, besides the purchasing costs and the impact of the generation of biological waste to the environment. Therefore, the preparation without the use of gloves, with bare hands, seems to be the ideal recommendation.
